# Basophils Predict Mite Sensitization in Patients with Kawasaki Disease

**DOI:** 10.3390/children10071209

**Published:** 2023-07-12

**Authors:** Ling-Sai Chang, Ying-Hsien Huang, Hsin-Yu Chang, Zon-Min Lee, Wei-Ling Feng, Ho-Chang Kuo

**Affiliations:** 1 Department of Pediatrics and Kawasaki Disease Center, Kaohsiung Chang Gung Memorial Hospital, Kaohsiung 833, Taiwan; 2 School of Medicine, College of Medicine, Chang Gung University, Taoyuan 333, Taiwan; 3 Department of Pharmacy, Kaohsiung Chang Gung Memorial Hospital, Kaohsiung 833, Taiwan; 4 Department of Pharmacy, Tajen University, Pingtung 907, Taiwan; 5 The Biostatistics Center, Kaohsiung Chang Gung Memorial Hospital, Kaohsiung 833, Taiwan

**Keywords:** basophils, immunoglobulin E, IVIG, Kawasaki disease, Kawasaki syndrome, specific IgE

## Abstract

**Background**: Patients with Kawasaki disease (KD) are at a significantly increased risk of allergic diseases. Immunoglobulin E (IgE) is an immunoglobulin that mediates allergic sensitization to various allergens and is related to various allergic diseases. However, few studies have analyzed specific IgE on allergy biomarkers after KD is diagnosed. **Objective**: This study aimed to investigate the pattern of specific IgE levels against food and inhalant allergens. **Methods**: This retrospective study was conducted in Taiwan to identify patients admitted with KD. A subset of 453 admitted KD children younger than or equal to five years of age with intravenous immunoglobulin (IVIG) was followed up at our clinic with available specific IgE data. **Results**: The most common allergens were Dermatophagoides farina or pteronyssinus, house-dust, and cockroach mix. Positive specific IgE for Dermatophagoides farina or pteronyssinus was less common in children diagnosed with KD who were two years old or younger (*p* = 0.028). KD patients with higher basophils before IVIG (*p* = 0.010 and 0.018 for two different mites) and higher C-reactive protein (CRP, *p* = 0.030 and 0.028) after IVIG were at higher risk of mite sensitization. Integrated mite sensitization demonstrated higher basophils before IVIG, age at KD diagnosis, and the male sex to be clinically meaningful after logistic regression models. **Conclusions**: This study is the first to suggest that specific IgE in KD patients may be correlated with age at KD diagnosis, as well as basophils. Further longitudinal prospective studies are warranted to clarify the unique profile of specific IgE in KD patients.

## 1. Introduction

Various data, including the activation of B cells and the analysis of B cell receptors, have suggested the role of infection by pathogens in Kawasaki disease (KD) [1,2]. Non-infectious triggers such as mites have also been reported as a causative agent in KD [3,4]. Real-world studies have pointed out that patients that received high-dose intravenous immunoglobulin (IVIG), whether to treat KD or severe enterovirus, may show a significant increase in eosinophils [5]. As a result, IVIG is also suspected to be one of the reasons for the increase in allergic diseases after KD [6]. The anti-inflammatory mechanisms of IVIG include saturating Fc receptors and inhibitory effects on the mRNA expression of Fc receptors, lowering the production of cytokines, and on epigenetic modulation to increase gene methylation [7,8]. Among patients treated with IVIG, only patients with hyper immunoglobulin(Ig) E syndrome or atopic dermatitis had decreased IgE levels after treatment, and levels in patients with KD or idiopathic thrombocytopenic purpura were unchanged [9]. Many drugs have complex effects on immunity, which can have both negative and positive effects on allergic inflammation [10]. IVIG has a powerful therapeutic effect on KD, and the few side effects are mostly mild and temporary [11,12]. During the infusion, IgE-mediated immediate reactions may occur [13]. After KD patients finished immunoglobulin, unlike IgA, G, or M, no significant changes were found in IgE [14]. However, another study on KD patients with or without coronary artery lesions had inconsistent results and showed higher IgE in the acute phase than in the convalescent phase [15]. At the same time, the role of IgE in KD was not like IgG, A, or M, which had predictive roles for outcomes [14,16]. In previous studies, IgE levels were once found to be higher in KD patients than in the control group at the acute stage [17]. However, these findings were inconclusive. In a recent study, Shen et al. identified decreased IgA and IgG in the KD group compared with the control group, but not IgE or IgM levels [18]. However, IgE concentrations cannot reflect those that have been combined with IgE receptors nor the role of specific IgE due to low sensitivity [19]. Due to the activation of B cells in KD and the increase in allergic diseases in KD patients, the role of IgE also needs to be further explored and analyzed [20]. Specific IgE may have a much greater significance to a certain allergen [21,22,23]. Even though allergic diseases play a very important role when following up patients with KD, no research has currently been conducted on the role of specific IgE against food and inhalant allergens in patients with KD [5,6].

We used data from a retrospective cohort to investigate the associations between specific IgE levels and IVIG treatment. These associations were also explored between markers and allergen sensitization.

## 2. Methods

### Specific Immunoglobulin E in Chang Gung Research Database

In this study, we enrolled Taiwanese patients with acute KD who were admitted to Chang Gung Memorial Hospital between 1 January 2001 and 26 June 2019. We extracted data from the medical claims of the Chang Gung Research Database (CGRD), which includes de-identified personal data on demographics (sex, age), disease diagnoses, pharmacy records, laboratory data, and examination reports. Specific IgE data have been integrated into CGRD since 2015 [24,25]. We enrolled participants diagnosed with KD (disease code of International Classification of Disease, Ninth Revision [ICD-9]:446 or ICD-10: M30) and laboratory records of specific IgE after KD diagnosis during follow-up at Kaohsiung Chang Gung Memorial Hospital. This retrospective study was reviewed and approved by our facility’s institutional review board in Kaohsiung, Taiwan (IRB number: 202001038B0).

**Figure 1:**
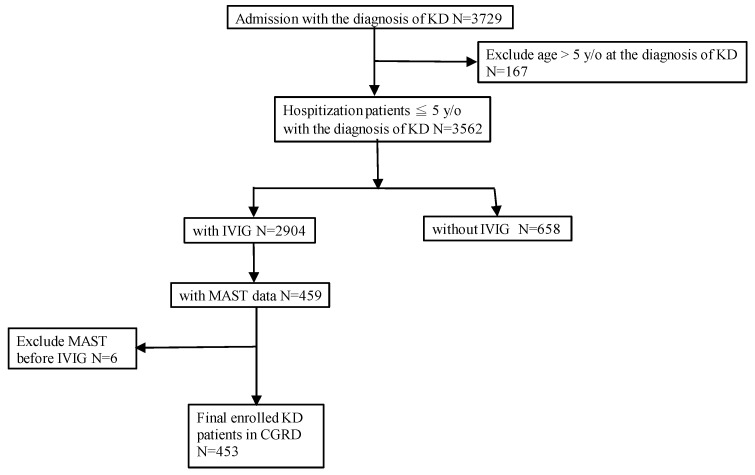
The study design of enrollment for the process of inclusion and exclusion of subjects. CGRD, Chang Gung Research Database; IVIG, intravenous immunoglobulin; KD, Kawasaki disease; MAST, multiple allergen simultaneous tests system; N, number; y/o, years old.

The basic information of subjects is listed in Table 1. Figure 1 shows the current study design of enrollment for the selection of subjects. We excluded inpatients with KD above five years old at the diagnosis of KD (*n* = 167). KD patients without IVIG treatment were excluded (*n* = 658). Subjects were excluded if they had tested for specific IgE prior to their KD diagnosis (*n* = 6). Patients with KD underwent specific IgE tests with 36 items (*n* = 453) using the validated multiple allergen simultaneous tests system (MAST, OPTIGEN; Hitachi Chemical Diagnostics, Inc., Mountain View, CA, USA) in Chang Gung Memorial Hospital. In this study, patients with positive results for specific IgE greater than or equal to one class constituted the positive specific IgE group; the remaining patients with zero class constituted the negative specific IgE group. We obtained and analyzed total IgE levels of the KD patients and performed specific IgE tests. Among KD patients with sIgE, 182 patients had at least one test by MAST.

All data are shown as mean with standard deviation. The CGRD results of the specific IgE collected from KD patients were analyzed and compared using *t*-test for continuous variables (Table 2) and Chi-square test for discontinuous variables. When comparing negative and positive groups for mite specific IgE at different time points, the *p*-values in Table 2 were corrected by a false discovery rate [26]. Logistic regression models were applied where the risk of positive specific IgE for mites was estimated. We performed statistical analysis using the Statistical Analysis System Package (SAS statistical software, Version 9.4; SAS Institute, Cary, NC, USA). A *p*-value of less than 0.05 was considered statistically significant.

## 3. Results

Among these patients, only one dose of IVIG accounted for approximately 86.8% of cases (393 of 453). Fifty-two patients required a second dose, and eight patients required a third dose. The follow-up period ranged 1595.2 ± 1476.3 days in KD patients with IVIG (Table 1). The IgE of patients who received IVIG twice or three times was not higher than that of KD patients who received IVIG only once (222.2 ± 321.8 vs. 312.2 ± 553.4 kU/L, *p* = 0.086).

The prevalence of positive specific IgE for allergens of inhalation (chicken feathers, Bermuda grass, black willow, eucalyptus, Japanese cedar, white mulberry, pigweed, ragweed mix I, Timothy grass, Alternaria, Aspergillus, Cladosporium, Penicillium, cat, dog, house-dust, cockroach mix, mite DF, and mite DP) or food (avocado, pork, beef, milk, cheddar cheese, shrimp, crab, clam, codfish, tuna, peanut, soybean, wheat, brewer’s yeast, egg yolk, and egg white) was 42.6% and 33.6% in KD patients with IVIG. In our study, approximately half of the KD patients with IVIG (54.5%) had an inhalant or a food sensitization.

The study of KD patients identified the five most common allergens as mite DF (Dermatophagoides farina, 38.9%), mite DP (Dermatophagoides pteronyssinus 37.3%), house-dust (21.2%), cockroach mix (15.5%), and beef (11.9%) in patients with IVIG.

We observed no significant difference in the sensitization of these 36 allergens between KD patients who received IVIG once and KD patients who received IVIG two or three times (*p* > 0.05) (Supplementary Table S1).

Most patients who contract KD are less than two years old (Table 1) [27]. In KD patients with IVIG diagnosed at more than two years old, the positive rate of the most positive allergen Dermatophagoides farina or pteronyssinus was higher than that of patients diagnosed with KD under the age of two years old (n = 28 positive for mite DF or DP in 51 patients diagnosed with KD older than 2 years old), and the class was higher (class 2 in five patients; class 3 or 4 in 19 patients) (Table 3). However, the other four top specific IgE (house-dust, cockroach mix, beef, and shrimp) did not differ significantly between KD patients diagnosed below the age of two years old and those above it (*p* > 0.05) (Table 3).

In Table 2, we analyzed KD patients who received IVIG treatment and found that the positive specific IgE for the most common allergens, mite DF or mite DP, was related to the C-reactive protein (CRP) within one week after IVIG (35.2 ± 37.8 and 26.4 ± 29.2 mg/L, *p* = 0.040). The albumin levels before and after IVIG treatment were lower, and the probability of positive specific IgE for mites in the future was also higher (mite DP 3.6 ± 0.6 and 3.8 ± 0.5 g/dL, *p* = 0.045 before IVIG; 3.2 ± 0.5 and 3.4 ± 0.4 g/dL, *p* = 0.018 after IVIG). A higher percentage of basophils before IVIG treatment was associated with being positive for mite specific IgE in the future (mite DF 0.3 ± 0.4 and 0.2 ± 0.3%, *p* = 0.010; mite DP 0.3 ± 0.4 and 0.2 ± 0.3, *p* = 0.018). Furthermore, a logistic regression model with variables including sex and age at diagnosis of KD was applied. Compared with KD patients not tested as having positive IgE for mites, those with positive IgE recorded a higher percentage of basophils (*p* = 0.004) according to age at KD diagnosis and male sex (*p* = 0.005 and 0.009, respectively).

In the initial screening panel, 27 patients who had a negative result in mite DF became positive, but 87 patients with a negative result still showed negative in the following MAST. In the initial screening panel, 25 patients who had a negative result in mite DP became positive, but 94 patients with negative result still showed negative in the following MAST. The characteristics of sIgE development for mites were compared. We found that the percentage of basophils after IVIG could predict the positive sIgE (DF, 0.34 ± 0.47 and 0.17 ± 0.27%, *p* = 0.0495; DP, 0.41 ± 0.51 and 0.17 ± 0.26%, *p* = 0.012) over time. The sIgE development for mites did not differ significantly from the negative group regarding age at first sIgE or KD, the time interval between the first and next tests, the number of IVIG, sex, albumin, alanine aminotransferas, aspartate transaminase, CRP, leukocytes, hemoglobulin, platelet, the percentage of eosinophils, lymphocytes, monocytes, and neutrophils before or after IVIG (*p* > 0.05). We found that the above factors did not predict the development of tolerance in mite allergy. In the initial screening panel, only 1 patient who had a positive result in mite DF became negative, but 67 patients with a positive result still showed positive in the following MAST. In the initial screening panel, 4 patients who had a positive result in mite DP became negative, but 59 patients with positive result still showed positive in the following MAST.

Interestingly, the percentage of eosinophils before and after IVIG treatment did not influence the positive rate of specific IgE for the top five positive allergens (*p* > 0.05). Furthermore, when the absolute eosinophil count before and after IVIG use was greater than 500/μL, it did not increase the positive rate of specific IgE for the top five allergens (mite DF or DP, house-dust, cockroach mix, beef, and shrimp) (*p* = 0.716, 0.832, 0.920, 0.628, and 0.184).

## 4. Discussion

We discovered that the top allergens in KD patients indeed matched the top allergens for allergic diseases in Taiwan [28]. In the database, the specific IgE tests were for patients with allergic symptoms. The positive rate of specific IgE was consistent with that of allergic patients aged 5–18 in Taiwan, and half of them tested positive for specific IgE [29].

Sensitization to mites had the highest population attributable risks for asthma, eczema, and rhinitis [23,29,30,31]. We found that the positive specific IgE for mites was associated with higher basophils before IVIG. Allergy is induced by the interaction between the allergens and IgE bound to mast cells and basophils that induce the release of inflammatory mediators [32]. Basophils initiate chronic allergic reaction and are essential for protease antigen specific IgE induction [33]. We also found that higher basophils of KD patients were likely to produce positive specific IgE for mites in the future. Since basophil is an important biomarker involved in atopic immune responses, these mechanisms may also be involved in the clinical progression of KD. The incidence of allergic diseases in children increases with age, and the addition of inflammation to the allergen sensitization process may explain the higher positive rate of specific IgE in KD diagnosed at older ages [28]. Glode et al. found no difference in anti-mite antibody concentrations between convalescent sera from KD and those from pediatric hospitalized controls [34].

We saw a wealth of research into allergic diseases and KD and how these were related to eosinophils, IgE, IgE receptors, and IL-4 [35,36,37,38]. A decade of research has now given us useful information on KD associated with allergic diseases [39,40]. At the same time, these allergic biomarkers have also been found to be useful in predicting the disease prognosis [41]. Given the limitations of the research method, our knowledge of basophils in KD diagnosis and patho-etiology was very limited. Seminal work on basophil histamine release in the diagnosis of mite allergy in asthmatic children was carried out by ØSTERGAARD (1990) and other researchers [42,43]. Our research has suggested potentially important influences of basophils on the sensitization of mites in KD patients.

CRP, neutrophils, and albumin have been commonly used parameters for measuring the activity of inflammatory conditions. [7] Previous reports have indicated that low serum albumin levels due to vasculitis and plasma leakage were correlated with nutrition and immune status and associated with IVIG treatment failure in KD patients, intensive care unit admission, and coronary artery lesions. Vascular leakage was suspected to not only result in hypoalbuminemia but also to allow allergens from the respiratory tract into the sub-epithelial space and circulation and further sensitization. Therefore, the inflammatory state, especially after IVIG treatment, reflecting the therapeutic effect of IVIG, would be related to the positive specific IgE for mites, rather than the number of IVIG administration.

Because the research of big data allowed us to follow up children with KD to the age when they would develop allergic diseases, we learned through cohort studies that asthma, rhinitis, and atopic dermatitis would increase after KD. The gradually increasing laboratory data in the CGRD were complementary to the shortcomings of the Taiwan National Health Insurance Research Database, which started to include laboratory data in 2021 [44].

Our study had several limitations. In this study, we did not determine specific IgE to be associated with KD outcomes. Resources to determine whether the patients had coronary artery dilatations were not available from the unstructured part of the CGRD system [45]. Because of the database design of this study, the absence of healthy controls or provocation tests in our study rendered a causal relationship between positive specific IgE and allergic symptoms unable to be concluded, and it was difficult to compare the positive rate of specific IgE between the controls and KD patients [46]. The major limitation of the current research was the retrospective design with missing data on laboratory parameters in enrolled patients with KD who underwent MAST (Table 2).

Our knowledge of characteristics of the specific IgE involved in KD children remains only partial. Further prospective studies are needed to address the many remaining questions concerning this issue.

## 5. Conclusions

To the best of our knowledge, the results of this cohort study are the first to demonstrate basophils contributed to mite sensitization among KD patients. In addition, the age of KD diagnosis and CRP were associated with mite sensitization. This finding lends support to the monitoring of allergic diseases and associated specific IgE for KD patients with higher basophils. According to the current study, specific IgE for mites has shown its importance for KD over time. This is the first study that followed food and inhalant allergen sensitizations after KD. Further longitudinal prospective studies are warranted to clarify the unique profile of specific IgE in KD patients.

## Figures and Tables

**Table 1:** Clinical data of the subjects in this study.

IVIG	Number	Age at Diagnosis of KD (Years Old)	Age at the Time of Specific IgE Test (Years Old)	Days between KD Diagnosis and Specific IgE Test	Female/Male
only one dose	393	1.5 ± 1.2	5.8 ± 4.0	1570.2 ± 1462.7	140/253
two or three doses	60	1.6 ± 1.1	6.4 ± 4.5	1579.0 ± 1565.3	23/37
*p* value		0.776	0.324	0.357	0.684

IgE, immunoglobulin E; IVIG, intravenous immunoglobulin; KD, Kawasaki disease.

**Table 2:** The sensitization of mites and laboratory parameters before (A,B) and (C,D) after intravenous immunoglobulin treatment in patients with Kawasaki disease. Combining specific immunoglobulin E of Dermatophagoides farina and pteronyssinus in Table 2E,F.

**(A)**				
**Specific IgE for Dermatophagoides Farina**				
**Before IVIG**	**N**	**Negative**	**Positive**	***p*** **Value**
WBC 10^3^/μL	424	13.8 ± 5.0	14.0 ± 5.2	0.772
PLATELET 10^3^/μL	424	349.0 ± 134.9	377.1 ± 143.1	0.676
HGB g/dL	424	11.0 ± 1.1	11.0 ± 1.2	0.826
CRP mg/L	424	80.9 ± 65.3	86.6 ± 72.6	0.401
NEUTROPHILS %	425	56.5 ± 15.6	60.0 ± 14.2	0.040 *
LYMPHOCYTE %	425	32.1 ± 14.4	29.1 ± 12.6	0.060
MONOCYTE %	425	6.5 ± 3.3	5.9 ± 2.9	0.098
EOSINOPHIL %	417	3.2 ± 3.1	3.4 ± 2.9	0.426
BASOPHIL %	403	0.2 ± 0.3	0.3 ± 0.4	0.010 *
ALB g/dL	357	3.8 ± 0.5	3.7 ± 0.5	0.125
ALT U/L	399	73.3 ± 100.4	73.3 ± 100.5	0.996
AST U/L	400	63.8 ± 87.8	69.1 ± 100.3	0.585
ESR mm/h	202	53.4 ± 24.4	52.6 ± 25.9	0.840
**(B)**				
**Specific IgE for Dermatophagoides Pteronyssinus**				
**Before IVIG**	**N**	**Negative**	**Positive**	***p*** **Value**
WBC 10^3^/μL	424	13.8 ± 5.1	13.9 ± 5.2	0.908
PLATELET 10^3^/μL	424	351.7 ± 135.6	373.1 ± 143.0	0.250
HGB g/dL	424	11.0 ± 1.1	10.9 ± 1.2	0.864
CRP mg/L	424	79.6 ± 64.5	88.8 ± 73.6	0.181
NEUTROPHILS %	425	56.4 ± 15.3	60.2 ± 14.6	0.024 *
LYMPHOCYTE %	425	32.2 ± 14.1	29.0 ± 13.1	0.044 *
MONOCYTE %	425	6.6 ± 3.3	5.7 ± 2.7	0.004 *
EOSINOPHIL %	417	3.1 ± 3.1	3.5 ± 2.9	0.172
BASOPHIL %	403	0.2 ± 0.3	0.3 ± 0.4	0.018 *
ALB g/dL	357	3.8 ± 0.5	3.6 ± 0.6	0.045 *
ALT U/L	399	75.9 ± 105.2	68.5 ± 90.9	0.894
AST U/L	400	65.7 ± 89.8	65.7 ± 97.4	0.997
**(C)**				
**Specific IgE for Dermatophagoides Farina**				
**After IVIG**	**N**	**Negative**	**Positive**	***p*** **Value**
WBC 10^3^/μL	389	10.5 ± 4.9	10.7 ± 4.4	0.772
PLATELET 10^3^/μL	389	471.3 ± 175.0	463.8 ± 161.8	0.676
HGB g/dL	389	10.7 ± 1.1	10.6 ± 1.2	0.826
CRP mg/L	367	26.3 ± 29.0	35.6 ± 38.3	0.030 *
NEUTROPHILS %	388	36.2 ± 16.6	38.6 ± 16.8	0.181
LYMPHOCYTE %	388	50.0 ± 15.9	48.5 ± 15.8	0.358
MONOCYTE %	388	7.5 ± 3.4	7.2 ± 2.9	0.346
EOSINOPHIL %	385	3.8 ± 3.1	4.3 ± 3.6	0.352
BASOPHIL %	369	0.3 ± 0.4	0.4 ± 0.5	0.022 *
ALB g/dL	273	3.4 ± 0.4	3.3 ± 0.5	0.125
ALT U/L	313	48.8 ± 64.8	50.0 ± 98.6	0.996
AST U/L	314	55.1 ± 68.6	46.2 ± 49.4	0.392
ESR mm/h	107	68.8 ± 33.1	72.0 ± 36.7	0.840
**(D)**				
**Specific IgE for Dermatophagoides Pteronyssinus**				
**After IVIG**	**N**	**Negative**	**Positive**	***p*** **Value**
WBC 10^3^/μL	389	10.5 ± 4.9	10.6 ± 4.3	0.908
Platelet 10^3^/μL	389	473.0 ± 174.4	450.1 ± 162.2	0.481
HGB g/dL	389	10.7 ± 1.1	10.7 ± 1.2	0.896
CRP mg/L	367	26.4 ± 29.2	36.0 ± 38.4	0.028 *
Neutrophils %	388	35.9 ± 16.8	39.2 ± 16.4	0.066
Lymphocyte %	388	50.3 ± 16.0	47.7 ± 15.5	0.122
Monocyte %	388	7.5 ± 3.5	7.2 ± 2.8	0.284
Eosinophil %	385	3.8 ± 3.1	4.4 ± 3.6	0.172
Basophil %	369	0.3 ± 0.4	0.4 ± 0.5	0.073
ALB G/DL	273	3.4 ± 0.4	3.2 ± 0.5	0.018 *
ALT U/L	313	49.5 ± 64.4	48.0 ±100.9	0.894
AST U/L	314	55.4 ± 68.2	44.9 ± 49.4	0.260
**(E)**				
**Specific IgE for Dermatophagoides Farina or Pteronyssinus**				
**Before IVIG**	**N**	**Negative**	**Positive**	***p*** **Value**
WBC 10^3^/μL	424	13.8 ± 5.0	13.9 ± 5.2	0.911
Platelet 10^3^/μL	424	350.4 ± 134.2	373.7 ± 144.2	0.182
HGB g/dL	424	11.0 ± 1.1	11.0 ± 1.2	0.941
CRP mg/L	424	80.9 ± 64.7	86.2 ± 73.0	0.431
Neutrophils %	425	56.4 ± 15.5	59.9 ± 14.4	0.040 *
Lymphocyte %	425	32.1 ± 14.2	29.3 ± 13.0	0.078
Monocyte %	425	6.6 ± 3.3	5.9 ± 2.9	0.052
Eosinophil %	417	3.2 ± 3.1	3.3 ± 2.9	0.568
Basophil %	403	0.2 ± 0.2	0.3 ± 0.4	0.008 *
ALB g/dL	357	3.8 ± 0.5	3.7 ± 0.6	0.138
ALT U/L	399	74.7 ± 101.6	71.0 ± 98.3	0.933
AST U/L	400	64.7 ± 89.0	67.4 ± 98.0	0.779
ESR mm/h	202	53.9 ± 24.1	51.7 ± 26.2	0.716
**(F)**				
**Specific IgE for Dermatophagoides Farina or Pteronyssinus**				
**After IVIG**	**N**	**Negative**	**Positive**	***p*** **Value**
WBC 10^3^/μL	389	10.5 ± 4.9	10.6 ± 4.4	0.911
Platelet 10^3^/μL	389	472.8 ± 176.0	461.4 ± 160.2	0.525
HGB g/dL	389	10.7 ± 1.1	10.7 ± 1.2	0.941
CRP mg/L	367	26.4 ± 29.2	35.2 ± 37.8	0.040 *
Neutrophils %	388	36.2 ± 16.7	38.6 ± 16.6	0.156
Lymphocyte %	388	50.1 ± 15.9	48.3 ± 15.7	0.278
Monocyte %	388	7.5 ± 3.5	7.2 ± 2.9	0.403
Eosinophil %	385	3.8 ± 3.1	4.3 ± 3.6	0.430
Basophil %	369	0.3 ± 0.4	0.4 ± 0.5	0.037 *
ALB g/dL	273	3.4 ± 0.4	3.3 ± 0.5	0.138
ALT U/L	313	49.4 ± 65.3	48.5 ± 96.8	0.933
AST U/L	314	55.4 ± 69.2	45.9 ± 48.5	0.326
ESR mm/h	107	69.1 ± 33.5	71.5 ± 36.1	0.716

ALB, albumin; ALT, alanine aminotransferase; AST, aspartate transaminase; CRP, C-reactive protein; ESR, erythrocyte sedimentation rate; HGB, hemoglobulin; IVIG, intravenous immunoglobulin; WBC, white blood cells. * statistically significant results (*p* < 0.05).

**Table 3:** Age at diagnosis of Kawasaki disease and the most common allergens.

		Number (%)	≤2 Year-Old	>2 Year-Old	*p* Value
Dermatophagoides Farina or Pteronyssinus	negative	269 (59.4)	246 (61.2)	23 (45.1)	0.028 *
	positive	184 (40.6)	156 (38.8)	28 (54.9)	
House-Dust	negative	357 (78.8)	320 (79.6)	37 (72.5)	0.246
	positive	96 (21.2)	82 (20.4)	14 (27.5)	
Cockroach Mix	negative	383 (84.5)	343 (85.3)	40 (78.4)	0.200
	positive	70 (15.5)	59 (14.7)	11 (21.6)	
Beef	negative	385 (85)	343 (85.3)	42 (82.4)	0.576
	positive	68 (15)	59 (14.7)	9 (17.6)	
Shrimp	negative	399 (88.1)	355 (88.3)	44 (86.3)	0.673
	positive	54 (11.9)	47 (11.7)	7 (13.7)	

* statistically significant results (*p* < 0.05).

## Data Availability

Data is unavailable because the owner of this database is Chang Gung Memorial Hospital.

## Supplementary Materials

The following supporting information can be downloaded at: https://www.mdpi.com/article/10.3390/children10071209/s1, Table S1: The sensitization pattern of allergens in patients with intravenous immunoglobulin (IVIG).
