# Supplementary material for: Basophils Predict Mite Sensitization in Patients with Kawasaki Disease
Source: Children (Basel). 2023 Jul 12;10(7):1209. doi: 10.3390/children10071209 (PMC10378518; doi:10.3390/children10071209)
Supplement: Supplementary file 1 [file children-10-01209-s001.zip › children-2460532-supplementary.pdf]

Table S1. The sensitization pattern of allergens in patients with intravenous immunoglobulin (IVIG).

	NUMBERS OF IVIG TREATMENT	NEGATIVE	POSITIVE	P VALUE
**DERMATOPHAGOIDES FARINA or PTERONYSSINUS**	one	236	157	0.458
	Two or three	33	27	
**DERMATOPHAGOIDES FARINA**	one	243	150	0.445
	Two or three	34	26	
**DERMATOPHAGOIDES PTERONYSSINUS**	one	249	144	0.453
	Two or three	35	25	
**HOUSE-DUST**	one	308	85	0.561
	Two or three	49	11	
**COCKROACH MIX**	one	332	61	0.917
	Two or three	51	9	
**DOG**	one	373	20	0.396
	Two or three	56	4	
**CAT**	one	381	12	0.574
	Two or three	58	2	
**PIGWEED**	one	384	9	0.440
	Two or three	58	2	
**BLACK WILLOW**	one	388	5	0.380
	Two or three	60	0	
**ALTERNARIA**	one	388	5	0.380
	Two or three	60	0	
**BERMUDA GRASS**	one	389	4	0.433
	Two or three	60	0	
**CLADOSPORIUM**	one	391	2	0.348
	Two or three	59	1	
**ASPERGILLUS**	one	390	3	0.497
	Two or three	60	0	
**EUCALYPTUS**	one	391	2	0.580
	Two or three	60	0	
**JAPANESE CEDAR**	one	390	3	0.497
	Two or three	60	0	
**WHITE MULBERRY**	one	392	1	0.696
	Two or three	60	0	
**RAGWEED MIX I**	one	391	2	0.580
	Two or three	60	0	
**TIMOTHY GRASS**	one	391	2	0.580
	Two or three	60	0	
**PENICILLIUM**	one	390	3	0.486
	Two or three	59	1	
**CHICKEN FEATHERS**	one	390	3	0.497
	Two or three	60	0	
**BEEF**	one	335	58	0.699
	Two or three	50	10	
**SHRIMP**	one	346	47	0.948
	Two or three	53	7	
**CRAB**	one	350	43	0.199
	Two or three	50	10	
**CLAM**	one	363	30	0.528
	Two or three	54	6	
**EGG WHITE**	one	365	28	0.807
	Two or three	57	3	
**PORK**	one	376	17	0.927
	Two or three	59	1	

**CHEDDAR CHEESE**	one	383	10	0.289
	Two or three	57	3	
**WHEAT**	one	380	13	0.867
	Two or three	59	1	
**TUNA**	one	383	10	0.487
	Two or three	58	2	
**PEANUT**	one	383	10	0.212
	Two or three	60	0	
**CODFISH**	one	384	9	0.625
	Two or three	58	2	
**SOYBEAN**	one	383	10	0.212
	Two or three	60	0	
**LATEX**	one	386	7	0.298
	Two or three	60	0	
**EGG YOLK**	one	386	7	0.298
	Two or three	60	0	
**MILK**	one	386	7	0.422
	Two or three	58	2	
**AVOCADO**	one	389	4	0.433
	Two or three	60	0	
**BREWER'S YEAST**	one	390	3	0.497
	Two or three	60	0	
